# Empowering biomedical learners to navigate FDA regulatory processes and entrepreneurship with a novel interdisciplinary training approach

**DOI:** 10.3389/fmed.2025.1522572

**Published:** 2025-02-06

**Authors:** Philip A. Cola, Tawna L. Mangosh

**Affiliations:** ^1^Department of Design, Innovation and Organizational Behavior, Weatherhead School of Management, Case Western Reserve University, Cleveland, OH, United States; ^2^Department of Population and Quantitative Health Sciences, School of Medicine, Case Western Reserve University, Cleveland, OH, United States; ^3^Department of Pharmacology and Center for Medical Education, School of Medicine, Case Western Reserve University, Cleveland, OH, United States

**Keywords:** interdisciplinary, transdisciplinary, innovations in biomedical education, regulatory science, entrepreneurship

## Abstract

For rising professionals to meet the needs of contemporary healthcare and biomedical innovation, educators must develop new teaching and learning approaches. Specifically, biomedical innovations are significantly influenced by the FDA’s regulatory framework, requiring professionals to be equipped with regulatory science knowledge, entrepreneurial skills, and interdisciplinary training. However, biomedical education often fails to integrate these skills in an environment that mimics the interdisciplinary setting required for translational science, leaving learners unprepared for unique challenges in practice. This study details an FDA Regulation and Entrepreneurship curriculum at Case Western Reserve University and its affiliated community, leveraging a novel approach for biomedical education. Focused on preparing biomedical professionals to navigate FDA regulatory processes and innovative entrepreneurship endeavors, the curriculum is built upon five core principles: integrating multiple disciplines, ensuring real world applicability, developing a systems thinking approach, incorporating ethical considerations, and fostering a collaborative and experiential learning environment. These principles are supported by a flexible course format, targeted learning objectives, team-based learning sessions, experiential learning opportunities, a diverse participant population, and an interdisciplinary team of faculty and experts. High participant engagement and broad representation across fields over the curriculum’s three-year lifespan to date affirm its relevance and value with participants representing the fields of basic science, medicine, law, business, and engineering. The flexible course format, team-based learning, and experiential learning proved instrumental in enhancing engagement, reinforcing practical learning outcomes, and supporting personalized learning goals. The flexible course format further aligns with professional needs of participants, providing a model for other institutions navigating similar challenges in biomedical education. In conclusion, participant feedback demonstrated the value of the interdisciplinary and transdisciplinary training approach in promoting knowledge retention and skill development in complex medical, business management and legal contexts. Moving forward, targeted outreach and flexible engagement options will be necessary to expand the curricular reach and diverse participant population. The success of the curriculum suggests promising implications for similar approaches aimed at empowering biomedical professionals with essential regulatory, entrepreneurial and interdisciplinary competencies, ultimately advancing translational science and improving healthcare outcomes.

## Introduction

Innovations in teaching and learning for biomedical educators have become increasingly necessary to address the evolving complexities of modern healthcare systems and biomedical innovation (1). As the landscape of biomedical education shifts toward more interdisciplinary and practice-oriented approaches, there is a growing need for educational models that not only teach core health sciences, but additionally equip learners with the skills to navigate regulatory frameworks and foster entrepreneurship in biomedical fields (2). In particular, the regulatory framework of the United States Food and Drug Administration (FDA) plays a critical role in determining the success of biomedical innovations, requiring biomedical professionals to be proficient in navigating its processes for drug, device, and biologic approvals. Yet, traditional biomedical education often lacks an integrative focus on these regulatory and entrepreneurial competencies, leaving graduates unprepared for the multifaceted challenges they face in practice (3). Therefore, this paper explores how interdisciplinary training can better prepare biomedical professionals for these challenges by integrating business management, law, engineering, and social sciences into biomedical education.

The problem of practice in higher education lies in the outdated curricular models that do not adequately address the interdisciplinary nature of modern healthcare innovation (4). Despite the increasing need for professionals to work across multiple domains, many educational programs remain siloed, with little collaboration between medical schools, business schools, and other relevant fields (5). The result is that learners graduate with a strong foundation in their discipline but lack the broader competencies required for success in complex adaptive systems, such as the FDA regulatory process (6). Compounding this issue, graduate enrollment in higher education has been on the decline over the past decade, with organizations less willing to invest in employees’ formal training. This trend presents a significant challenge for universities aiming to stay relevant while also meeting the needs of their learners and the demands of the healthcare industry (7).

Existing literature on interdisciplinary education in health professions and the biomedical sciences highlights the importance of integrative learning models, such as team-based learning (TBL), mentorship, and digital tools, which have been shown to improve student engagement, critical thinking, and collaboration (8, 9). However, there are notable gaps in these models, particularly in their ability to effectively bridge the divide between medicine and management (10). While case studies and isolated programs have demonstrated the value of interdisciplinary approaches, there remains a lack of comprehensive curricula that provide sustained, practical training in both regulatory science and biomedical entrepreneurship. Additionally, current models tend to focus on either the academic or the professional aspect of training, rather than offering a holistic approach that integrates real-world application with academic rigor (11).

This paper seeks to address these gaps by presenting a case study of a newly developed interdisciplinary curriculum that integrates medicine, basic science, business management, law, and engineering at a tier-1 research university. By evaluating data from three completed interdisciplinary courses, this study will examine the impact of innovative teaching strategies, such as TBL, case study analysis, and experiential learning on participant competencies in navigating FDA regulatory processes and fostering biomedical innovation (12). Furthermore, the curriculum offers flexibility in its delivery, allowing participants from various disciplines and professional backgrounds to earn informal certificates or micro-credentials and to earn formal transcriptable credits. The stakeholders most impacted by this work include prospective participants, biomedical educators, curriculum developers, policymakers, and healthcare organizations seeking to cultivate leaders capable of driving innovation in the field.

We hypothesize our interdisciplinary education model will better prepare biomedical professionals to navigate regulatory frameworks and drive biomedical innovation in complex healthcare environments. This work is highly relevant to the ongoing challenges faced by higher education institutions and biomedical educators in preparing learners to promote healthcare innovation. By offering student data-driven recommendations for enhancing interdisciplinary training, this paper contributes to the broader discourse on how to innovate teaching and learning practices in a way that aligns with the needs of both learners and the healthcare industry.

## Pedagogical framework and principles

The development of this innovative teaching and learning approach for regulatory science and biomedical innovation began with five core principles: (1) Integrating multiple disciplines; (2) Ensuring real world applicability; (3) Developing a systems thinking approach; (4) Incorporating ethical considerations encountered with interdisciplinary thinking; and (5) Fostering collaborative and an experiential learning environment (Table 1). Under this framework, this study set out to combine perspectives from medicine, science, engineering, business management, law, and regulatory science to provide a comprehensive understanding of the FDA approval process and biomedical entrepreneurship. The idea is to not only educate learners seeking this theoretical knowledge, but to ensure an emphasis on practical, hands-on learning through real world experiences of experts, case studies, TBL exercises and interactions with industry professionals (13, 14).

**Table 1:** Core principles guiding curriculum development and resulting learning objectives.

Core principles
A. Integrate multiple disciplines
B. Ensure real world applicability
C. Develop a systems thinking approach
D. Incorporate ethical considerations for interdisciplinary thinking
E. Foster collaborative and an experiential learning environment
Learning objectives
1. Summarize the history and mission of the FDA
2. Describe the drug approval process
3. Distinguish between different routes of product approval*
4. Compare and contrast biological product issues
5. Compare and contrast device and diagnostic approval process*
6. Discuss the importance of required clinical trial design elements
7. Identify the key principles for ethical human clinical testing*
8. Discuss ethical considerations related to generic medications
9. Identify FDA enforcement and post-marketing issues
10. Formulate a recommendation following IND/IDE review*
11. Synthesize knowledge gained to prepare an IND/IDE application*

The five core principles guiding the development of the regulatory sciences and entrepreneurship course offered to diverse participants including CWRU students, faculty, staff and others in affiliated hospital systems. This resulted in the development of 6 original learning objectives for AY 21–22 and the addition of 5 learning objectives for AYs 22–24; *Learning objectives added for AYs 22–24.

The aforementioned principles were developed in advance of the course as aspirational goals to ensure innovation and relevant experiences for students and working professionals. To develop this curriculum in accordance with contemporary needs for biomedical innovation, it is necessary to develop a systems thinking approach to global healthcare needs (4). This would allow participants to think critically about real world problems of practice in a transdisciplinary manner to generate new ideas for innovative solutions (15). This is the general understanding that the world is not linear and it requires a systematic approach, complete with recursive feedback loops, to contribute to the most challenging problems in biomedical innovation (16). Additionally, the incorporation of discussions on various legal and ethical implications of biomedical innovations and the appropriate regulatory approaches to ensure compliance without inhibiting idea generation was necessary. Finally, the core principle of fostering teamwork among participants from diverse academic and professional backgrounds to best mirror real world interdisciplinary collaborations was essential. Therefore, innovative curriculum design and pedagogical approaches were developed to support these principles during the course.

While this framework primarily adopts an interdisciplinary approach, it is important to distinguish this from a transdisciplinary approach which is also incorporated but to a lesser degree. Interdisciplinarity requires the integration of knowledge and methods from different disciplines internal and external to medicine in this context. However, this historically maintains the clear boundaries between disciplines and the awareness that you are crossing those boundaries. This approach is valuable as it helps to then focus more on synthesizing these diverse perspectives across the learning process. However, pedagogically the plan required a higher level of integration or transdisciplinary elements to fully support the core principles. This transcends those traditional disciplinary boundaries and blurs the typical differentiation between alternative perspectives (17). Ideally, this creates a more unified framework that goes above and beyond individual disciplines to create something where integration is of a higher order compared to the individual components. Under these circumstances, participants will be able to create new conceptual, theoretical and methodological approaches. This higher order level is essential for catalyzing the translation of discoveries from basic science laboratories to testing in humans, to testing in patients, then to the practice of medicine and ultimately to the community of practice.

In the context of this course, an interdisciplinary approach allows learners from various backgrounds (i.e., medicine, nursing, engineering, business management, basic sciences, etc.) to bring their unique perspectives while learning to collaborate. A transdisciplinary approach then goes further to potentially develop entirely new frameworks for understanding biomedical innovation that transcend existing disciplinary structures. This framework emphasizes interdisciplinary science and learning to ensure learners gain a comprehensive understanding while still respecting the distinct contributions of each field. However, elements of transdisciplinarity are incorporated to encourage participants to develop innovative solutions or new pathways that might challenge traditional disciplinary boundaries.

## Learning environment

### Curriculum oversight

Interdisciplinary curriculum addressing regulatory science and entrepreneurship was developed at Case Western Reserve University (CWRU) as a joint effort between the School of Medicine (SOM) and Weatherhead School of Management (WSOM) by curriculum leaders from each respective school. The advisory committee formed to support this initiative assisted curriculum leaders with the development of learning objectives, teaching approaches, curricular materials, and experiential learning opportunities to support the pedagogical framework principles outlined above. The resulting course was offered annually for three consecutive academic years (AYs).

### Target audience

Course advertisements were sent to undergraduate and graduate students in relevant programs as well as the faculty and staff supporting those relevant departments and programs across CWRU. Notably, the course was also advertised to the four affiliated hospital systems in the Greater Cleveland Area. Specifically, The Cleveland Clinic, University Hospitals, MetroHealth, and Cleveland Veteran Affairs Medical Center. Targeted advertisements were sent to members of the Case Comprehensive Cancer Center and Clinical and Translational Science Collaborative. Prospective participants were encouraged to share advertisements with others in their professional networks, not affiliated with CWRU, but interested in the content and experiential opportunities.

### Course format evolution

The format of the course began as an informal certificate course consisting of 10 two-hour sessions in AY 21–22. Synchronous, in-person and virtual options were offered to allow hybrid delivery and support our diverse participants. Interested faculty, staff, and other non-student participants were required to pay a registration fee and would receive a certificate of completion following successful attendance at 80% or more sessions. To incentivize healthcare provider participation, continuing medical education (CME) credit was offered for each session. Additionally, several slots with waived registration were offered to students on a first come, first serve basis. The AY 21–22 sessions primarily adopted a flipped classroom, discussion-based approach with limited lecture-based sessions delivered by experts in their respective field, in line with the framework outlined above and course learning objectives (Table 1).

To further accommodate our diverse participants comprising the target audience and address the needs of the CWRU and affiliated community, the course format in AY 22–23 was converted to a formal, flexible credit hour course for students with the informal certificate course still offered for non-student participants. Based on low in-person attendance the previous year, the hybrid course was converted to a virtual-only format. Fourteen, two-hour sessions were now included, 10 of which retained the original format described above. Remaining sessions focused on TBL, leveraging case studies based on real world experiences of the experts lecturing and leading discussions.

TBL is a strategy that requires participant engagement in pre-class preparation, in-class individual and team assessments, and team application exercises to promote active learning and collaboration (18, 19). For this course, teams were thoughtfully assigned to ensure participants from different disciplines (i.e., medicine, business management, law) were grouped together. This approach allowed teams to leverage the collective knowledge of the team to tackle real-word case studies (Supplementary Figure S1), mimicking the interdisciplinary and transdisciplinary collaboration needed to navigate the regulatory process and entrepreneurial ventures (20, 21).

The formal flexible credit hour designation allowed students to take the course for either one or three transcriptable credits to count toward elective requirements for their degree program. Students enrolled in the course for one credit were expected to attend at least 80% of the sessions and complete a reflection as a follow up to each TBL session (Supplementary Figure S1). Pedagogically it is important to encourage reflection on teamwork, personal growth, and understanding while learning from others in the classroom (5, 18, 19). As an authentic assessment, those enrolled for three credits also completed two projects outlining components required for an Investigational New Drug (IND) or Investigational Device Exemption (IDE) application for a technology already approved (midterm; Supplementary Figure S2) and a hypothetical technology in line with their research interests (final; Supplementary Figure S3) (22). Differences in assigned student activities allowed for justification of the flexible credit hour format. Those opting for the informal certificate option had similar attendance and registration requirements as above. CME credit was still offered to incentivise healthcare provider participation.

### Learning objectives and course content

Based on the core principles outlined above and the collaborative effort of the curriculum leaders and advisory committee, eight original learning objectives were developed to guide participant learning in regulatory science and entrepreneurship. These were adopted for the informal certificate course launched in AY 21–22. For AYs 22–24, five learning objectives were added to align with the additional sessions and assignments needed for formal flexible credit, especially those focused on IND or IDE application assignments (Table 2). To ensure alignment of course content and learning objectives, each session and assignment was mapped to relevant learning objective(s) and core principle(s) (Table 2).

**Table 2:** Course sessions and assignments supporting core principles and learning objectives.

Session	Session topic and relevant assignment or project	Speaker or grader field of expertise	Mapped core principle	Mapped learning objective
1	History of FDA and FDA Mission	Medicine, Law, Science, FDA	A-B, E	1
2	Inside the FDA: Reflections of a Former Division Director	Medicine, FDA	A-C, E	1–2
3	The Drug Approval Process	Medicine, Law, Science, FDA	B-C, E	1, 3
4	Regulatory Considerations for Biologics	Management, Science, FDA	B-C, E	1–4
5	Team-based Learning Session 1	All Fields	A-E	1–4
	*Assignment*: Written Reflection 1	All Fields	A-E	1–4
6	Human Clinical Testing	Management, FDA	A-E	1–3, 6, 7
7	Clinical Trial Design Issues	Medicine, FDA	B-D	1–3, 6, 7
8	Generic Drugs and Ethics	Science, FDA, Engineering	B, D	1–3, 8
9	Team-based Learning Session 2	All Fields	A-E	1–8
	*Assignment*: Written Reflection 2	All Fields	A-E	1–8
10	FDA Review Process	Medicine, Science, FDA	A-C, E	1, 10, 11
11	FDA Enforcement	Medicine, Law, Science, FDA	A-D	1, 9, 10
	*Project*: IND/IDE Outline Approved Product	All Fields	A-E	1–11
12	Post-marketing Issues	Medicine, Science, FDA	B-D	1, 9
13	Medical Devices and Diagnostics	Science, FDA, Engineering	A-C, E	1–3, 5
14	Team-based Learning Session 3	All Fields	A-E	1–11
	*Assignment*: Written Reflection 3	All Fields	A-E	1–11
	*Project*: IND/IDE Outline Hypothetical Product	All Fields	A-E	1–11

Course outline indicating session number, session topic with relevant assignment or project, and session speaker or assignment grader field of expertise. Additionally, core principles and learning objectives from Table 1 were mapped to each session.

The primary goal of the course and associated content was to support the learning and application of regulatory science and entrepreneurship to support inter- and transdisciplinary collaboration and healthcare innovation. To achieve this in an experiential, interdisciplinary learning environment, the basic FDA regulatory process was introduced early in the course (23, 24). Then medical and biological product development with legal and ethical considerations was presented and discussed (25). This provided the fundamental staging from concept to market, an overview of the clinical trial process, the institutional review board process, and steps to data submission review and approval. Finally, entrepreneurial fundamentals including disclosure, protection of intellectual property and licensing was introduced from a business management perspective that included market analyses and funding strategies (26). These ideas were developed against the backdrop of regulatory principles and strategic approaches to regulatory management. This helps learners craft effective regulatory submissions and communication plans (26). The latter has to be understood to develop skills needed to bridge knowledge gaps between disciplines while practicing techniques for ideation, prototyping and iterative human centered design principles in healthcare.

Faculty and guest speakers from diverse fields, including but not limited to physician scientists, business and legal experts, and regulatory and commercialization experts, were recruited to deliver course content that aligned with their expertise and to share real-world problems and perspectives from their field (Table 2). To further strengthen the team of experts delivering course content, biotechnology industry experts and successful entrepreneurs were recruited to deliver relevant sessions and further provide opportunities to explore entrepreneurial skills critical for biomedical professionals. Additionally, the involvement of industry experts provided an unique mentorship opportunity for course participants to grow their professional network of support.

### Course evaluation

Course evaluation and a continuous quality improvement plan is needed to ensure the diverse groups of course participants achieve the learning objectives and competencies needed to navigate the regulatory process and entrepreneurship. Additionally, an evaluation plan will ensure our course adapts to the ever evolving biomedical innovation landscape and needs of the CWRU and Greater Cleveland community. A requirement for all courses or sessions offered as CME credits is an anonymous survey completed by participants after course completion. Leveraging this requirement, course participants were asked to complete an anonymous post-course survey each AY. Survey questions focused on participant demographics, achievement of course objectives, satisfaction with TBL activities, and an open text box to collect qualitative feedback. Retrospective analysis of survey data collected as part of the CME accreditation process was deemed exempt from IRB review by the CWRU IRB (STUDY20240761).

## Results

### Participant diversity mimics interdisciplinary environment of biomedical innovation

One of the core principles guiding the development of this course was integrating multiple disciplines in order to support inter- and transdisciplinary experiential learning mimicking the diverse environment promoting healthcare innovation. While the curriculum design primarily supported interdisciplinary learning, it was important to establish a diverse group of course participants from varied training backgrounds and disciplines. Specifically, participants from medicine, law, business management, engineering and other disciplines would support the interdisciplinary interactions in TBL and discussion-based sessions necessary to support the remaining core principles of the course. To recruit diverse participants, recruitment strategies outlined above were used and demographic data collected from surveys and course registration forms was compiled and analyzed each year to confirm successful recruitment. In AY 21–22, 22–23, and 23–24, the number of participants who registered for the course was 40, 33 and 45, respectively. Importantly, nearly 100% of participants each year attended at least 80% of the sessions resulting in the awarding of a certificate of completion. Similarly, all students enrolled for formal credit received passing grades.

**Figure 1:**
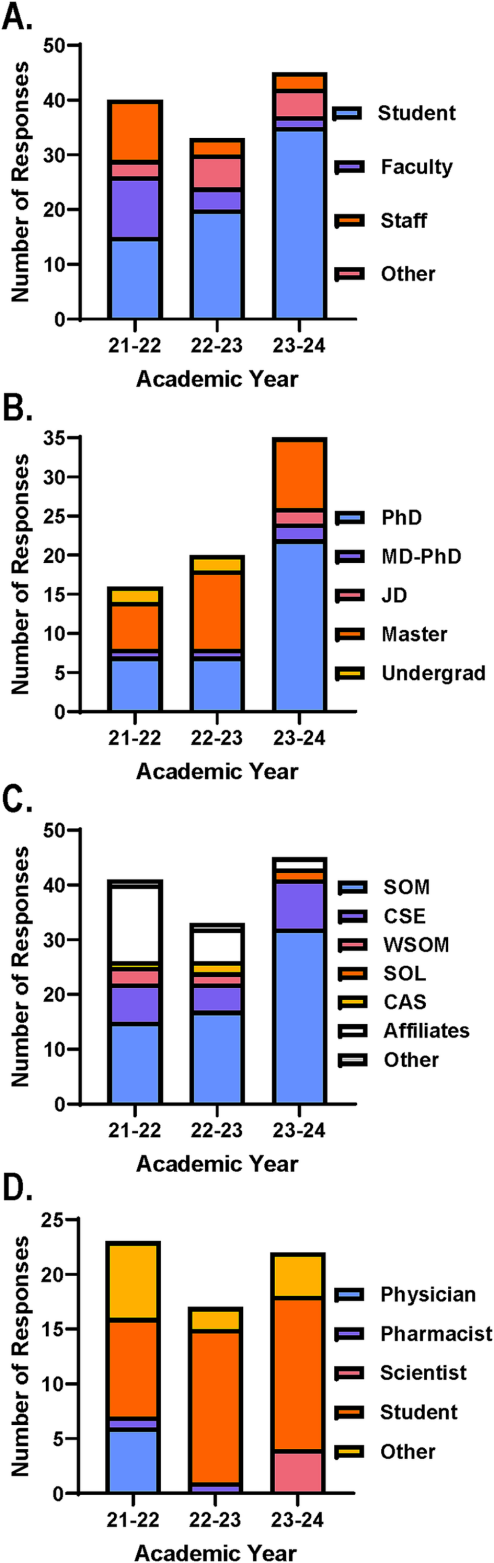
Course and survey participant distribution across years ensures interdisciplinary experiential learning environment. Course participant distribution based on number of registration form responses across AYs. Participants were categorized by **(A)** position, **(B)** student degree type, **(C)** associated school, college, unit, or affiliated hospital system. SOM, School of Medicine; CSE, Case School of Engineering; WSOM, Weatherhead School of Management; SOL, School of Law; CAS, College of Arts and Sciences. **(D)** Survey participant distribution based on number of responses on end of course surveys across AYs. Participants were categorized by position defined by CME requirements.

Participants were categorized as students, faculty, or staff at CWRU or affiliated hospital systems (Figure 1). Participants that did not fall in these categories included primarily postdoctoral fellows or individuals in leadership positions and were included in the other category. Interestingly, the participant distribution shifted from being relatively balanced across student, faculty and staff groups in AY 21–22 to demonstrating higher student representation in following years (Figure 1A). This effect coincided with creating a formal flexible credit option and more widespread student advertisement.

Further analysis of the student participants revealed that students from various training levels and fields were interested in the course across AYs offered (Figure 1B). The greatest number of participants identified as either PhD or master students, which included students pursuing an MS or MBA. The remaining participants were either undergraduate students with a biomedical-related major, graduate students pursuing a JD, or graduate students pursuing a dual degree of an MD and PhD (Figure 1B). Moving forward, advertisement and recruitment strategies will support the continued diversity in student participants interested in our course with targeted opportunities to reach others from the minimally represented degrees (i.e., MD-PhD, JD, undergraduate).

Participants were also categorized by associated units, such as CWRU school or college. Those who were part of the CWRU community but were not associated with a specific unit were categorized as other and those who were a part of one of the affiliated hospital systems were grouped together as affiliates (Figure 1C). While the majority of participants were associated with the School of Medicine (SOM) and the Case School of Engineering (CSE) via the Biomedical Engineering Program, it was encouraging to see representation from other units across campus and affiliated hospital systems (Figure 1C). Specifically, Weatherhead School of Management (WSOM), School of Law (SOL), and the College of Arts and Sciences (CAS) had associated participants enroll during at least 1 year the course was offered. In the future, efforts will be made to ensure continued, yet expanded participation from units and affiliates already represented and those not yet participating.

With regard to the anonymous course evaluation survey distributed at the end of the course, the response rates were ~ 58%, ~52% and ~ 49% for AYs 21–22, 22–23, and 23–24, respectively. As per CME requirements, participants completing the survey indicated if they were a physician (i.e., MD, DO, MD-PhD), pharmacist (i.e., PharmD), scientist (i.e., PhD, MS), student, or other (Figure 1D). Just as before the other group primarily consisted of postdoctoral fellows. Notably, the number of physicians completing the survey dwindled over the AYs the course was offered which likely corresponds to the reduced number of course participants hailing from the affiliated hospital systems over the same years (Figure 1D). This is important to note to allow for additional support and strategies to recapture prospective participants from these affiliates for future course installments.

### Achievement of personal goals and competencies by diverse participants

**Figure 2:**
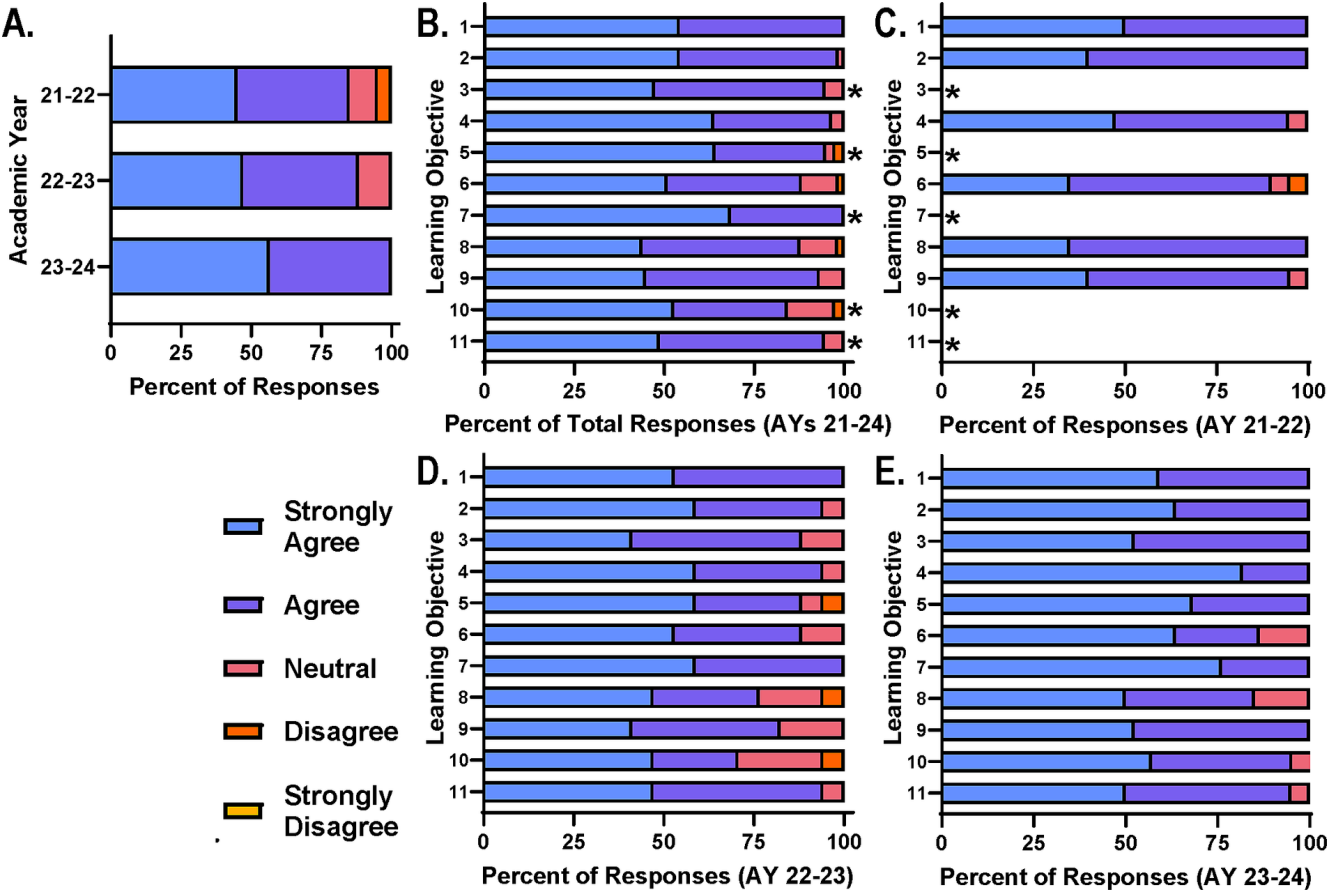
Participants agree course design and delivery supports achievement of learning objectives. **(A)** Course participants were asked on end of course surveys to rate their level of agreement with: “My personal learning objectives were met” on a scale of strongly agree to strongly disagree. Participants were also asked to provide their level of agreement with: “The course learning objectives were met” on a scale of strongly agree to strongly disagree. **(B)** Total responses for each learning objective across AYs, **(C)** AY 21–22, **(D)** AY 22–23, and **(E)** AY 23–24 are reported for comparison; *Learning objective added after AY 21–22.

Following the successful recruitment and participation of a diverse participant cohort each year, it was important to identify course components that were most successful in supporting the course goals and core principles, specifically from the participant perspective. Additionally, trends across years in participant perceptions and feedback will provide additional clarity. Participants were first asked to rate their level of agreement with the course’s ability to support the achievement of their own personal learning objectives (Figure 2A). Encouragingly the majority of participants agreed that their personal objectives were met through the course. Only a few participants felt as if they either disagreed or were neutral toward this statement in AY 21–22 but this diminished with each passing year, possibly due to the incorporation of new learning opportunities in AY 22–23 and the refinement of those in AY 23–24 (Figure 2A).

With regard to specific course learning objectives, participants were similarly asked to rate their level of agreement with the course’s ability to support the achievement of each objective (Figures 2B–E). Collectively, survey data across AYs 21–24 suggest that the majority of participants agree that the course supports the learning objectives with only a few participants disagreeing or feeling neutral toward the statement (Figure 2B). This pattern was consistent across survey data from each year when analyzed independently (Figures 2C–E). It is important to note that learning objectives 3, 5, 7, 10, and 11 (Table 1) were added for AY 22–23 to further support the core principles. Based on survey data, refinement of the sessions and activities supporting these objectives was needed (Figure 2D) and the number of those in agreement increased as a result for AY 23–24 (Figure 2E). This refinement included adjustment of topic schedule, TBL case study edits, team-teaching of specific topics and additional guidance for projects.

### Enhanced interdisciplinary experiential learning through team-based learning

**Figure 3:**
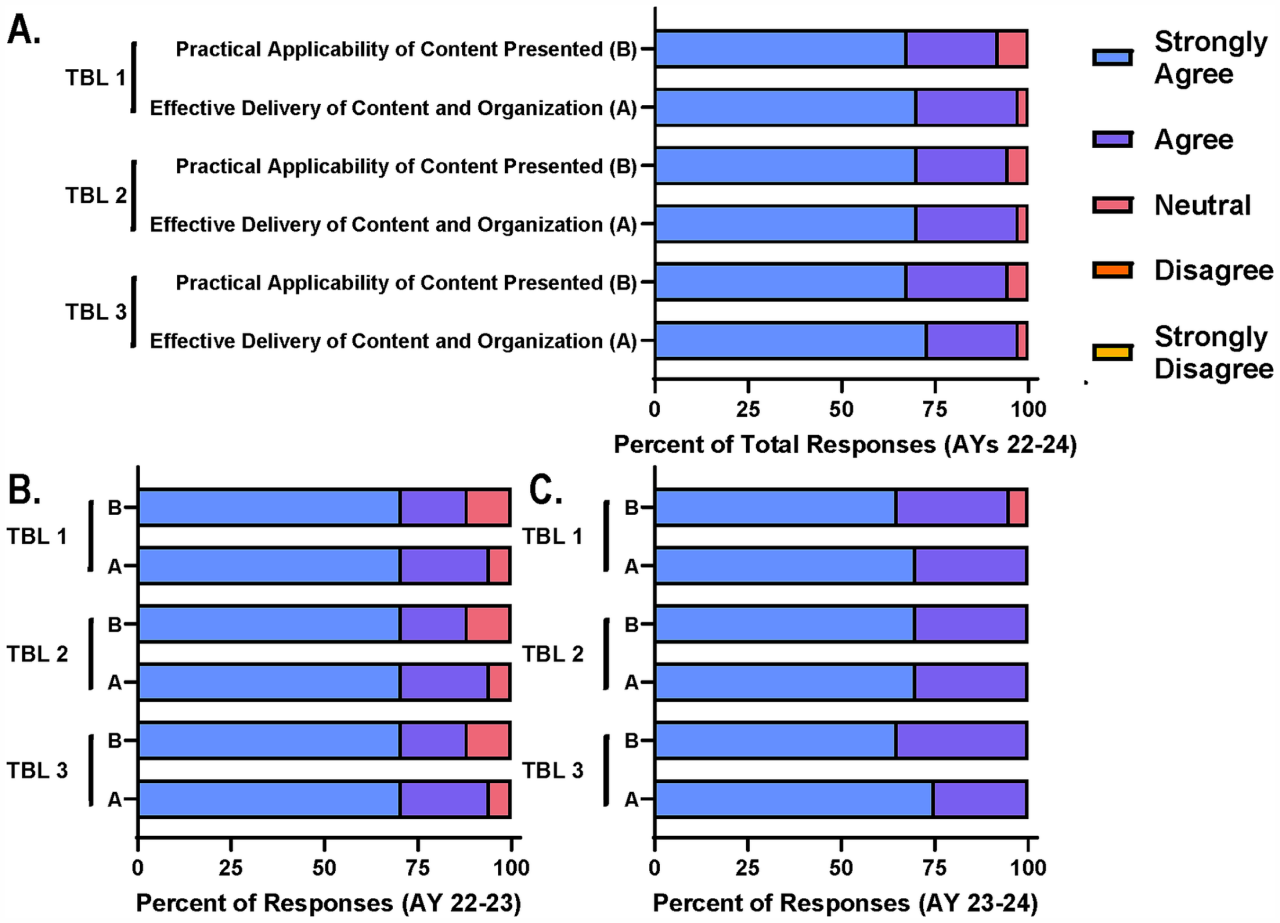
Participants agree TBL sessions support achievement of course goals and interdisciplinary collaboration. Course participants were asked on end of course survey to rate their level of agreement with two statements related to the practical applicability and effective delivery of content through TBL sessions. **(A)** Total responses for each TBL session across AYs, **(B)** AY 22–23, and **(C)** AY 23–24 are reported for comparison.

As mentioned above, three TBL sessions were incorporated into the schedule for AY 22–23 and AY 23–24 as part of the course changes that justified the formal flexible credit option. Specifically, TBL sessions for this course included pre-reading assignments before the session, readiness assurance discussions to begin the session, and two to three case studies designed by experts and based on real world situations. Each case study was accompanied by several probing questions to guide and encourage discussion among the interdisciplinary groups assigned (Supplementary Figure S1). Each group would read the case study and discuss the questions in a breakout room via Zoom and come to a consensus statement or stance which they would then report out to the larger group in the main Zoom meeting room. These sessions were designed in a way to support all core principles and the achievement of most learning objectives for the courses. As part of the survey distributed at the end of the course in AYs 22–24, participants were asked to rate their level of agreement with the TBL’s ability to support the practical applicability of course content and effective delivery of content and organization (Figures 3A–C). Again, the majority of participants agreed with these statements with the number of limited participants disagreeing or responding as neutral dwindling from AY 22–23 (Figure 3B) to AY 23–24, coinciding with session refinement efforts (Figure 3C).

### Innovative curricular design achieves course goals and objectives

Overall these data support that our recruitment and advertising strategies are able to successfully recruit diverse participants to support the inter- and transdisciplinary goals of the course. While there will always be room for improvement in representation from diverse disciplines, participants’ diversity was sufficient to support the core principles, promote achievement of personal and course learning objectives, and ensure the success of TBL sessions. Moreover, the format, content and delivery by diverse speakers supports the achievement of personal and course learning objectives. Finally, survey data support the use of TBL sessions to reinforce the core principles and provide an opportunity for the effective delivery and applicability of course content in an interdisciplinary and collaborative group setting.

## Discussion

These findings highlight the effectiveness of an interdisciplinary and transdisciplinary approach to teaching and learning for biomedical education, specifically in preparing participants for the complexities of FDA regulatory processes and biomedical entrepreneurship. The high participant engagement and diversity over three AYs indicate sustained importance of content and skills taught as well as the relevance to a broad audience of current or future basic science, healthcare, law, business management, and engineering professionals. The inclusion of students, faculty, and staff across schools at CWRU, but also from affiliated hospital systems demonstrates similar interdisciplinary educational models are capable of mimicking the environment of and cultivating the skills necessary to foster biomedical innovation. Furthermore, these findings suggest that the recruitment strategies employed were successful in attracting diverse participants that align with and support the course goal of collaborative learning across disciplines. Leveraging this approach and core principles, two additional courses in this space entitled *Patent Law and the Biomedical Sciences* and *Regulatory Strategy and FDA Communication* were created. Moving forward, this educational approach will facilitate the creation of additional curricula designed to solve complex interdisciplinary challenges.

The recruitment of diverse faculty, FDA regulatory experts, and biomedical entrepreneurs to deliver the course content further supported the course goals of collaboration across disciplines. From the content experts conducting the class came diverse real-world experiences that were converted into TBL sessions in AY 22–23. These sessions required experiential and collaborative learning in interdisciplinary groups which further enhanced participant engagement and reinforced the practical applicability of content and skills. As a result, the majority of participants reported the course successfully supported their personal learning objectives, with feedback improving each year as course elements were added and refined. The progressive alignment of course learning objectives with content, especially with the addition of new objectives, sessions, and authentic assessments in subsequent academic years, reflects responsiveness of course developers to participant feedback and commitment to continuous quality improvement. This trend and participant feedback indicates that experiential learning paired with interdisciplinary collaboration can significantly improve engagement as well as knowledge retention and application in complex regulatory and entrepreneurial contexts.

Despite course popularity among the CWRU community and affiliates, a decrease in participation from healthcare affiliates was observed over time, particularly among physicians. Though this decline coincides with increased student-targeted advertising and enrollment in AYs 22–24, this trend highlights the need for targeted recruitment efforts to re-engage this important population. The current and future physician and physician-scientist population is crucial given the importance of regulatory knowledge in research and clinical practice. To address this, future plans include asynchronous learning options, expanded CME credit opportunities, and collaborations with affiliated hospital systems to encourage greater participation. Recruitment and advertising plans also include improved outreach to students in the MD program at CWRU who historically enrolled in this course significantly less than students from other degree programs. The course described herein has already demonstrated significant flexibility to aid participant engagement from AY 21–22 to AY 23–24 and will be able to continually adjust to meet the needs of the course, participants, and the biomedical industry. For example, successful implementation of flexible credit options and micro-credentialing allowed participants from various fields and levels of training to engage in the way that best fit their current professional needs and future career aspirations. These future efforts will maintain strong representation from all relevant fields and ensure the interdisciplinary benefits are maximized, which is essential for understanding the multifaceted nature of transdisciplinary biomedical innovation and regulatory frameworks.

Overall, the findings of this study contribute valuable insights into the design of biomedical education that effectively integrate regulatory, entrepreneurial, and interdisciplinary competencies. The TBL sessions and interdisciplinary group discussions accentuate the need for such to become the standard for biomedical education going forward. Future iterations of this curriculum could build on these insights, exploring additional strategies to enhance interdisciplinary representation and continuously adapt based on the evolving industry and participant feedback (27). This approach could serve as a model for other educational institutions aiming to remain strong and relevant in an era where traditional degree programs have not fully met industry needs, which is becoming more important with declining graduate program enrollment. The successes described herein underscores the need for related or similar curriculum to prepare healthcare, FDA regulatory, and scientific professionals for the complex, adaptive systems they will encounter as necessary stepping stones to advance innovation within healthcare. Ultimately, such advances in biomedical education will catalyze the translational science paradigm, thereby improving healthcare outcomes (28).

## Data Availability

The raw data supporting the conclusions of this article will be made available by the authors, without undue reservation.

## References

[1] Woolliscroft JO. Implementing biomedical innovations into health, education, and practice: preparing tomorrow’s physicians Academic Press (2019).

[2] Clark K Hoffman A. Educating healthcare students: strategies to teach systems thinking to prepare new healthcare graduates. J Prof Nurs. (2019) 35:195–200. doi: 10.1016/j.profnurs.2018.12.006, PMID: 31126396

[3] Eisenberg R. The role of the FDA in innovation policy. Michigan telecommunications and technology law review (2007). Available at: https://www.semanticscholar.org/paper/The-Role-of-the-FDA-in-Innovation-Policy-Eisenberg/b1cf644045eb55110bc393c8af30369c930d1a74 (Accessed September 24, 2024).

[4] Frenk J Chen L Bhutta ZA Cohen J Crisp N Evans T . Health professionals for a new century: transforming education to strengthen health systems in an interdependent world. Lancet. (2010) 376:1923–58. doi: 10.1016/S0140-6736(10)61854-5, PMID: 21112623

[5] Hall P Weaver L. Interdisciplinary education and teamwork: a long and winding road. Med Educ. (2001) 35:867–75. doi: 10.1046/j.1365-2923.2001.00919.x, PMID: 11555225

[6] Weaver L McMurtry A Conklin J Brajtman S Hall P. Harnessing complexity science for interprofessional education development: a case study. J Res Interprof Pract Educ. (2011) 2:100–120. doi: 10.22230/jripe.2011v2n1a48, PMID: 27813256

[7] Hersh RH Merrow J. Declining by degrees: higher education at risk. New York: Palgrave Macmillan (2005) Available at: https://archive.org/details/isbn_9781403973160 (Accessed September 22, 2024).

[8] Barry ES Grunberg NE. Team medicine in an inter-professional environment In: Quinn JF White BAA, editors. Cultivating leadership in medicine. Dubuque, IA: Kendal Hunt Publishing Company (2019).

[9] Mukhalalati B Elshami S Eljaam M Hussain FN Bishawi AH. Applications of social theories of learning in health professions education programs: a scoping review. Front Med. (2022) 9:912751. doi: 10.3389/fmed.2022.912751 PMC9367215 35966845

[10] Quinn JF Cola P. Understanding physician leadership: the mediating effects of positive organizational climate and relational role endorsement. J Bus Ind Mark. (2020) 35:1491–503. doi: 10.1108/JBIM-01-2019-0032

[11] Dienstag JL. Relevance and rigor in premedical education. N Engl J Med. (2008) 359:221–4. doi: 10.1056/NEJMp0803098, PMID: 18635426

[12] Kolb DA. Experiential learning: experience as the source of learning and development Pearson Education Ltd (2014) Available at: https://ptgmedia.pearsoncmg.com/images/9780133892406/samplepages/9780133892406.pdf (Accessed September 20, 2024).

[13] Abdulwahed M Nagy ZK. Applying Kolb’s experiential learning cycle for laboratory education. J Eng Educ. (2009) 98:283–94. doi: 10.1002/j.2168-9830.2009.tb01025.x

[14] Austin M Rust D. Developing an experiential learning program: milestones and challenges. Int J Teach Learn Higher Educ. (2015) 27:143–53.

[15] Hopkins MM Ibanez F Skingle M. Supporting the vital role of boundary-spanning physician researchers in the advancement of medical innovation. Future Healthcare J. (2021) 8:e210–7. doi: 10.7861/fhj.2021-0091, PMID: 34286187 PMC8285151

[16] Batt A Brydges M Leyenaar M Tavares W. New ways of seeing: supplementing existing competency framework development guidelines with systems thinking. Adv Health Sci Educ. (2021) 26:1355–71. doi: 10.1007/s10459-021-10054-x, PMID: 34003391

[17] Hoffmann-Longtin K Kerr AM Shaunfield S Koenig CJ Bylund CL Clayton MF. Fostering interdisciplinary boundary spanning in health communication: a call for a paradigm shift. Health Commun. (2022) 37:568–76. doi: 10.1080/10410236.2020.1857517, PMID: 33289430

[18] Burgess A van Diggele C Roberts C Mellis C. Team-based learning: design, facilitation and participation. BMC Med Educ. (2020) 20:461. doi: 10.1186/s12909-020-02287-y, PMID: 33272267 PMC7712595

[19] Flores-Cohaila JA Moreno Ccama VP Baca Quispe AL Lopez Ayquipa AM Paz Gamarra FA Alfaro Peña PV . The constituents, ideas, and trends in team-based learning: a bibliometric analysis. Front Educ. (2024) 9:1458732. doi: 10.3389/feduc.2024.1458732

[20] Freeth R Caniglia G. Learning to collaborate while collaborating: advancing interdisciplinary sustainability research. Sustain Sci. (2020) 15:247–61. doi: 10.1007/s11625-019-00701-z

[21] Pazos P Pérez-López MC González-López MJ. Examining teamwork competencies and team performance in experiential entrepreneurship education: emergent intragroup conflict as a learning triggering event. Educ Train. (2022) 64:461–75. doi: 10.1108/ET-06-2021-0208

[22] Sokhanvar Z Salehi K Sokhanvar F. Advantages of authentic assessment for improving the learning experience and employability skills of higher education students: a systematic literature review. Stud Educ Eval. (2021) 70:101030. doi: 10.1016/j.stueduc.2021.101030

[23] Ga VN. Drugs, devices, and the FDA: part 1: an overview of approval processes for drugs. JACC Basic Transl Sci. (2016) 1:170–9. doi: 10.1016/j.jacbts.2016.03.002, PMID: 30167510 PMC6113160

[24] Van Norman GA. Drugs, devices, and the FDA: part 2: an overview of approval processes: FDA approval of medical devices. JACC. (2016) 1:277–87. doi: 10.1016/j.jacbts.2016.03.009 30167516 PMC6113340

[25] Darrow JJ Avorn J Kesselheim AS. FDA approval and regulation of pharmaceuticals, 1983-2018. JAMA. (2020) 323:164–76. doi: 10.1001/jama.2019.20288, PMID: 31935033

[26] Hanel P. Intellectual property rights business management practices: a survey of the literature. Technovation. (2006) 26:895–931. doi: 10.1016/j.technovation.2005.12.001

[27] Emanuel EJ. The inevitable reimagining of medical education. JAMA. (2020) 323:1127–8. doi: 10.1001/jama.2020.1227, PMID: 32105294

[28] Faupel-Badger JM Vogel AL Austin CP Rutter JL. Advancing translational science education. Clin Transl Sci. (2022) 15:2555–66. doi: 10.1111/cts.13390, PMID: 36045637 PMC9652430

