# Supplementary figures and images for: Empowering biomedical learners to navigate FDA regulatory processes and entrepreneurship with a novel interdisciplinary training approach
Source: Front Med (Lausanne). 2025 Feb 6;12:1522572. doi: 10.3389/fmed.2025.1522572 (PMC11839641; doi:10.3389/fmed.2025.1522572)

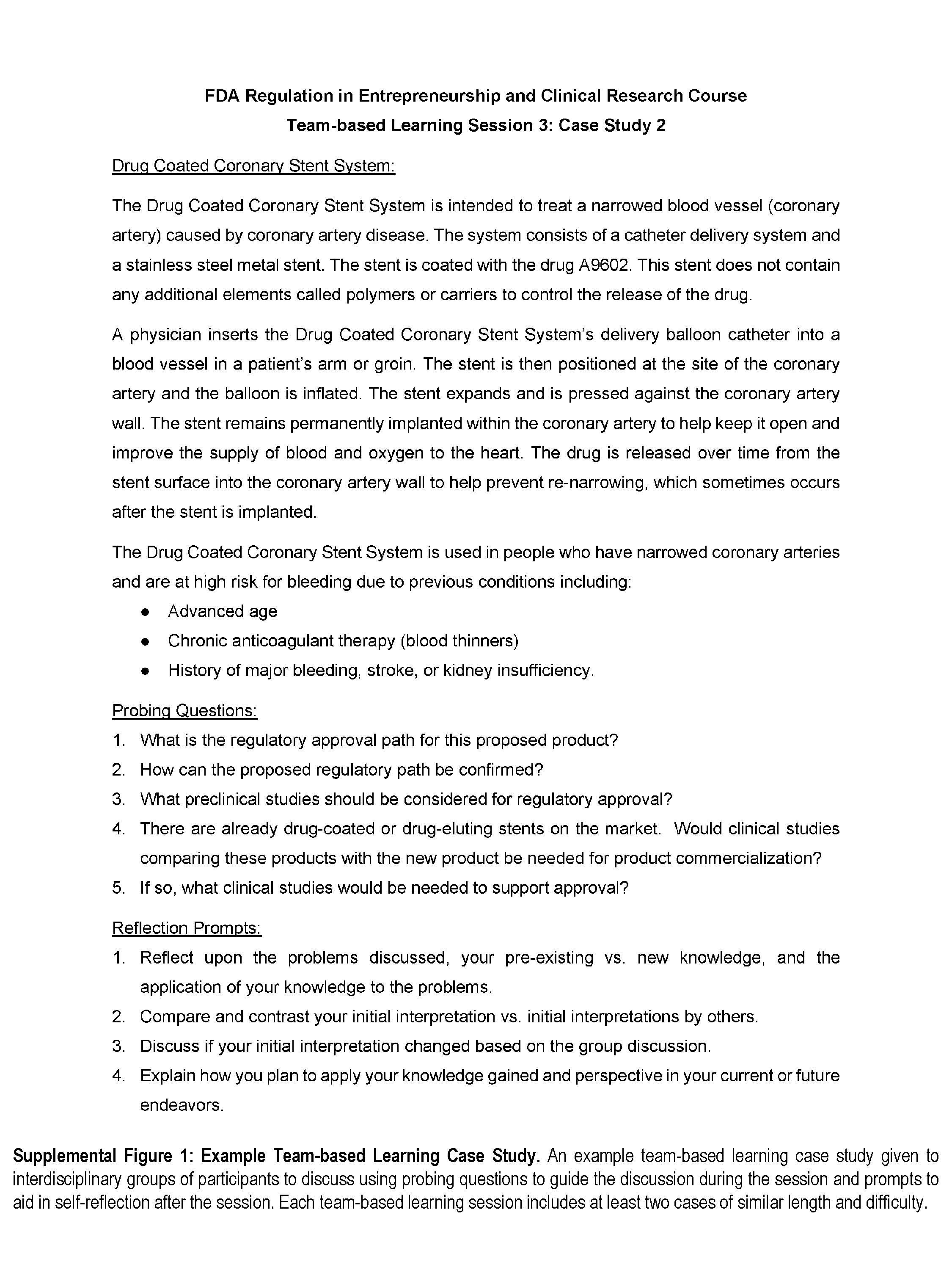

Supplement: Supplementary file 1 [file Image_1.TIF]

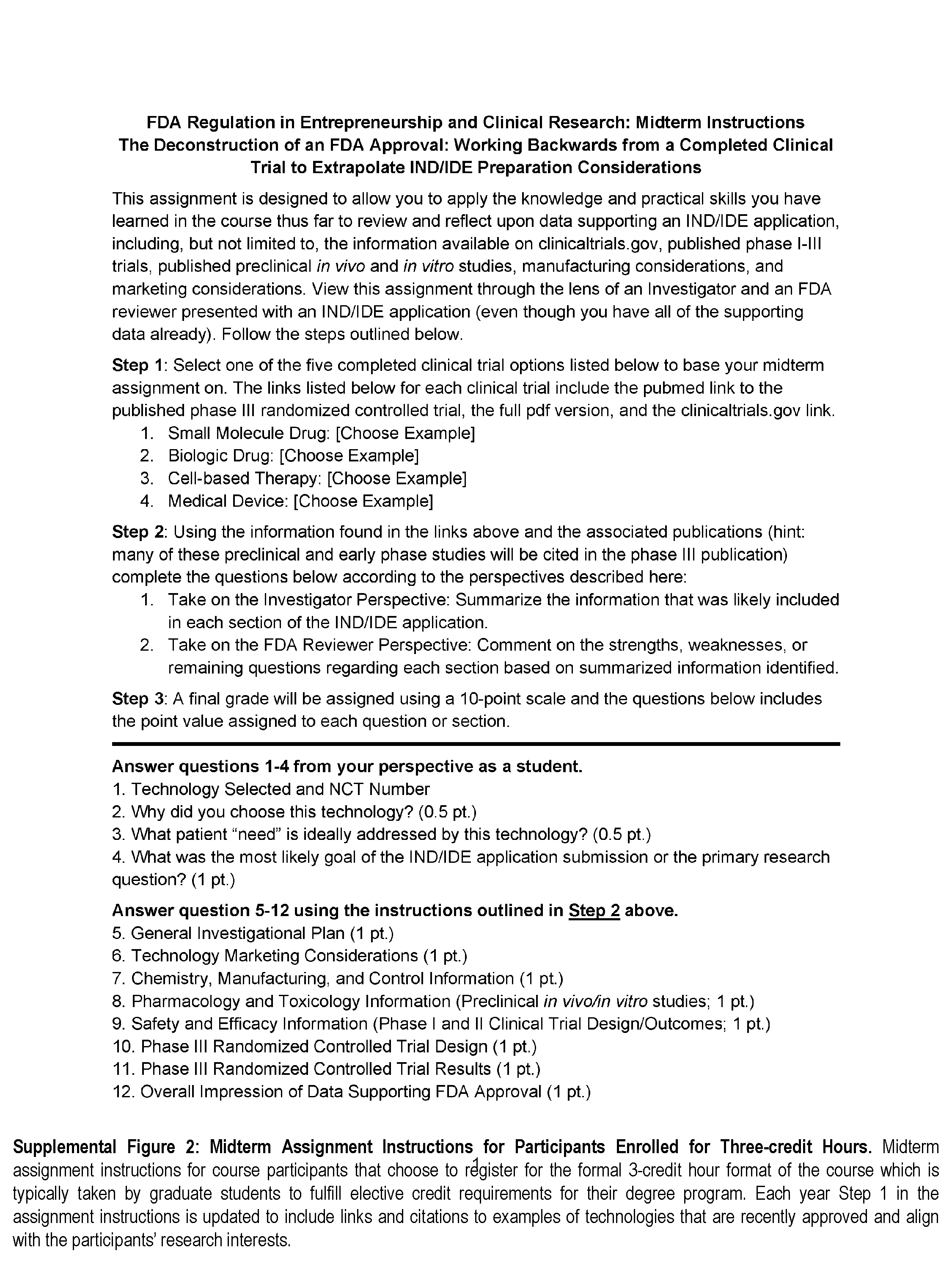

Supplement: Supplementary file 2 [file Image_2.TIF]

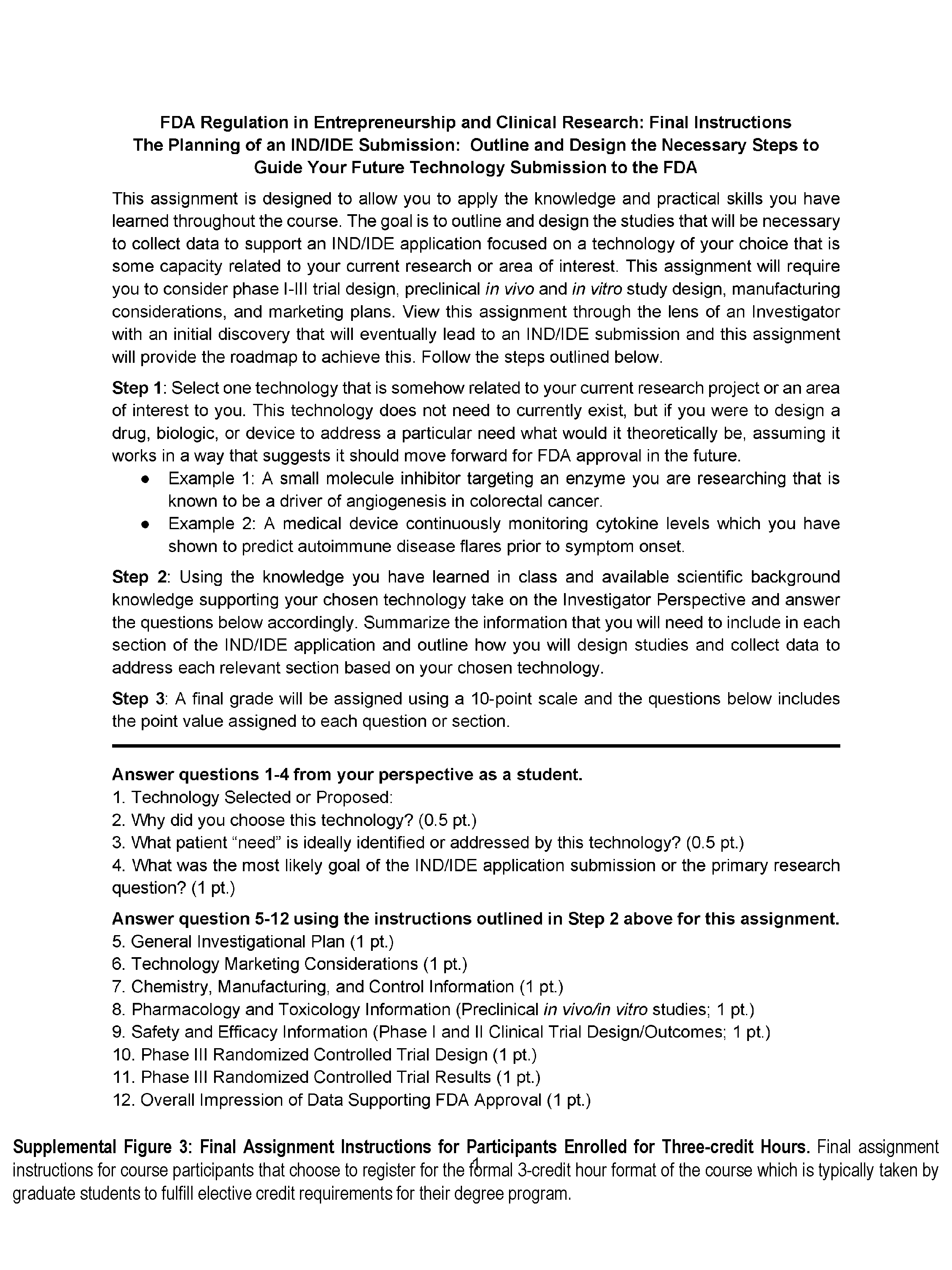

Supplement: Supplementary file 3 [file Image_3.TIF]
